# Embedding clinical trials in clinical practice: Harvesting experience and winnowing evidence

**DOI:** 10.4103/0253-7613.68416

**Published:** 2010-08

**Authors:** Arun S. Nanivadekar

**Affiliations:** Independent Medical Research Consultant, Mumbai - Pune, India E-mail: asnanivadekar@hotmail.com

In this essay I wish to present a personal perspective of clinical research, especially as relevant to medical pharmacologists.

**Regulatory trials:** Phase 3 trials as a regulatory requirement are formally over as soon as a new drug is approved for marketing. They provide fairly precise answers to a few questions, but not necessarily to many questions that concern doctors, patients, and payers.[1] These unanswered questions are relevant to the spectrum of patients in whom the drug may or could be used. Therefore, the real trial of a drug, where it would either stand or fall, begins *after* it is marketed.

**Shortcomings:**The gap between the data that are enough to approve a drug and the data needed to define its proper place in therapeutics results from three causes.

First, phase 3 trials focus mainly on surrogate endpoints which can be reached within a reasonable time, and which seem logically to bear on the ultimate desired outcomes, although there is no guarantee that this will always or necessarily be so.[2] Further, they may lead to other, unforeseen ultimate outcomes which may be either useful or harmful. Examples of such surrogate endpoints are: fall in blood pressure, blood sugar, or blood lipids; increase in serum titers of antibodies against specific micro-organisms; and inhibition of one but not another of a class of enzymes such as cyclo-oxygenases. However, the outcomes desired by doctors and patients are different, e.g., prevention of stroke, myocardial infarction, renal failure, or an infectious disease in those at risk. Besides, in addition to the benefit-to-risk ratio, the payers also want to know the benefit-to-cost ratio. To assess how a drug fares with these two ratios would require a much larger number of patients to be followed up for a much longer time and would also cost a great deal more.

Second, phase 3 trials include patients with strict selection and exclusion criteria, with the result that they form a sample that is not representative of all the patients in whom the drug may or could be used. Their generalizability is limited. To expand it, patients with much less restrictive criteria, and therefore with many more concomitants, need to be treated and evaluated. This would also cost a great deal more. The large number of mega trials carried out during the last decade with antihypertensive, antithrombotic, and hypolipidemic drugs are eloquent examples. Many of them have used composite endpoints—e.g., occurrence of death, or stroke, or nonfatal MI, all bundled together—which reduce the number of patients required for detecting a significant difference, but whose clinical importance, and hence relevance to clinical practice, becomes questionable.[3]

Third, the conditions in which a doctor may find a drug useful, either through serendipity or through pathophysiological and pharmacological reasoning, may be of only topical interest or of insufficient commercial potential for its manufacturer. In such cases, the doctor himself or herself will need to be the sponsor-investigator of the idea, and carry out the research in his or her practice.[4,5] Such practices were the cradle in which clinical research was born and grew until the industrialization of drug research and its regulation by governments scaled up its volume and cost, and took over the shaping of its goals.[6,7] There have been several fallouts of these developments:(1) the cost of drug discovery and development has approached the mark of 1 billion US dollars; (2) the conceptual contribution of doctors and patients to clinical research has dwindled; (3) a feeling has formed that clinical research is too costly, complex, and cumbersome to be done in day-to-day practice; and (4) several therapeutic uses of drugs continue to be branded “off-label” even though there has been enough experience of their rationale and benefits which can be winnowed for adequate evidence to verify them. Examples like the two I have cited above have not yet created enough impetus among doctors to undertake research in their practices on health care issues that directly concern them, and current regulations are probably fettering rather than fostering such research.[8] In the UK, Chalmers has at least openly voiced this concern.[9] In our country, there seems to be little awareness of it.

**Need for doctor scientists:**In his 1948 presidential address to the Section of Experimental Medicine and Therapeutics of the Royal Society of Medicine, Pickering[10] persuasively argued the importance of grooming doctors in the experimental method rather than feeding them “facts,” the inherently experimental nature of any branch of therapeutics, the need for testing even logically sound ideas through experiments, and the feasibility of a medical man being both a good doctor and a good scientist. “For if we take a patient afflicted with a malady, and we alter his conditions of life… by administering to him a drug, or by performing on him an operation, we are performing an experiment. And if we are scientifically minded we should record the results.” Practicing doctors are treating patients daily, and thus performing experiments, but perhaps not recording the results systematically. The information they are generating continuously is just being lost like unattended crop. It needs to be harvested and winnowed for evidence to address questions of genuine therapeutic uncertainty. What makes such an endeavor eminently possible now is the current state-of-the-art of information technology, especially the feasibility and affordability of digitizing numerical, textual, audio, and visual data for storage, transmission, pooling, processing, and analysis.

Peto[11] has been emphasizing the need for large simple trials for quite some time. He believes that current randomized controlled trials collect 10 to 100 times more data than are really needed.[12] The trials he proposes could be successfully embedded in day-to-day practice, collecting limited but relevant data, not only on a large number of patients but also over a long period of time. For such an endeavor, what would be needed?

**Two-pronged strategy:** Today’s students are tomorrow’s doctors. During their graduation course, they should be taught not only the facts and current concepts, but also the *methods* by which they were discovered and developed. To those going in for specialization, a foundation course in research methodology must be taught, and linked with their dissertation, so as to groom them as future investigators. I can think of two suitable resources for this. (1) a booklet published in 2001 which, despite its title, is an excellent primer of clinical research.[13] (2) the free software Epi Info,[14] which combines epidemiological tutorials, relational database designing, statistical analysis, and graphics. Medical pharmacologists can take a lead in developing such a program for all specialties in their institution, and running it jointly with teachers of clinical subjects. This is the first prong. I cannot resist the temptation of stressing the importance of this effort by quoting from the booklet I just referred to:

“The purpose of research is to create the knowledge essential for action to improve health. Without this knowledge, action is impossible because it has no logical or empirical basis. Indeed, ongoing action for health, if it does not contain an imbedded program of research, frequently becomes irrelevant, misleading, or unnecessarily costly…”

“Research is an activity of perpetual questioning. While public health practice is based on consensus, standardisation, and systematic practice, research requires a skeptical mind, prepared to continuously evaluate and question. This questioning and evaluating, when put into a systematic framework, creates the new knowledge that is required to create and continually modify actions for health. This is what research is and why it is important.“

The second prong would be the creation of *networks* of research-oriented practices. This could begin with a listing of practicing doctors who are willing to examine one or two therapeutic issues that interest or concern them, and helping them form networks to address those issues or concerns. For this purpose also, Epi Info can be used to develop customized applications, and deploying them for uniform data collection and management. The program developed for postgraduate students can be offered to the participating doctors on a web platform. We should systematically strive to develop and nurture research-oriented doctors in all branches of practice. Only then can certain inquiries be pursued over a long time to generate large, representative, standardized databases containing “just enough” data about patients, interventions, outcomes, and relevant concomitants. It would then be possible to winnow such harvested experience for evidence, either for or against a hypothesis, making allowance for various confounders, and creating sound evidence. A glimpse of the potential of such networks can be gleaned from a recent proposal for cohort multiple randomised control trials.[15] Besides, clinical research in practice need not be limited to drugs alone; it could also encompass prevalence and incidence, diagnostics, devices, prophylactic and therapeutic strategies, and non-drug therapies.

**The challenge:**Pharmacologists with a medical background, as well as physicians working in drugs, diagnostics, and devices industries, seem eminently equipped for the task outlined above through collaboration among academics, industry scientists, medical professionals, and regulators. Will they rise up to this challenge? I hope they do.
